# Supplementary material for: A registered report on how implicit pro-rich bias is shaped by the perceiver's gender and socioeconomic status
Source: R Soc Open Sci. 2020 Aug 26;7(8):191232. doi: 10.1098/rsos.191232 (PMC7481720; doi:10.1098/rsos.191232)
Supplement: Supplemental Analyses [file rsos191232supp1.docx]

**SUPPLEMENTAL MATERIAL FOR ROYAL SOCIETY OPEN SCIENCE REGISTERED REPORT:**

**A registered report on how implicit pro-rich bias is shaped by the perceiver’s gender and socioeconomic status**

Bradley D. Mattan^1^ & Jasmin Cloutier^2^

Affiliations:

^1^ Annenberg School for Communication, University of Pennsylvania, Philadelphia, PA, USA

^2^ Department of Psychological and Brain Sciences, University of Delaware, Newark, DE, USA

Corresponding author:

Bradley D. Mattan

Annenberg School for Communication

University of Pennsylvania

3620 Walnut St.

Philadelphia, PA 19104

Telephone: +1 (312)-450-9194

Email: [brad.mattan@gmail.com](mailto:brad.mattan@gmail.com)

# Supplemental Analyses 1: Parallel Regressions

## Pilot Dataset

**Personal evaluations.** In the model examining the participant’s own attitudes toward the rich versus poor, we observed a significant Income × Education interaction, *b* = 0.5926, *SE* = 0.2159, *CI_95%_ =* [0.166, 1.019], *t*(155) = 2.745, *p* = .007. At low and average education levels, increasing income was associated with reduced explicit pro-rich bias, *b* = -1.185, *SE* = 0.399, *CI_95%_ =* [-1.972, -0.397], *t*(155) = -2.972, *p* = .003. At high education levels, this pattern appeared to reverse, but this was non-significant, *b* = 0.593, *SE* = 0.367, *CI_95%_ =* [-0.132, 1.318], *t*(155) = 1.616, *p* = .108. At low income levels, increasing education was associated with reduced pro-rich bias, *b* = -1.172, *SE* = 0.406, *CI_95%_ =* [-1.973, -0.371], *t*(155) = -2.891, *p* = .004. At high income levels, this pattern appeared to reverse, but this was non-significant, *b* = 0.606, *SE* = 0.359, *CI_95%_ =* [-0.105, 1.316], *t*(155) = 1.684, *p* = .094. In summary, the greatest pro-rich explicit bias was observed for individuals with the lowest income and education, which is the opposite of what we found in our analyses of IAT data. In addition to this Income × Education interaction, we also observed a non-significant Gender × Education interaction, *b* = -0.791, *SE* = 0.410, *CI_95%_ =* [-1.601, 0.018], *t*(155) = -1.931, *p* = .055. This non-significant interaction was characterized by an apparent decrease in pro-rich bias with increasing education levels, but only for men, *b* = -0.679, *SE* = 0.335, *CI_95%_ =* [-1.341, -0.017], *t*(155) = -2.025, *p* = .045, and not women, *b* = 0.112, *SE* = 0.236, *CI_95%_ =* [-0.353, 0.578], *t*(155) = 0.476, *p* = .635. All other effects in this analysis of the participant’s self-reported evaluations of the poor versus rich were non-significant, *p* > .07.

**Ambivalence.** In the model examining the participant’s ambivalence toward the poor versus rich, we observed a significant main effect of education, *b* = 0.084, *SE* = 0.040, *CI_95%_ =* [0.005, 0.163], *t*(56) = 2.118, *p* = .039. Greater education was associated with a stronger mixture of positive and negative feelings toward the rich compared to the poor. This is intriguing because greater education was also associated with generally greater pro-rich implicit bias. In other words, greater ambivalence may not necessarily correspond to a reduction in pro-rich implicit bias. In addition to this main effect of education, we also observed a significant Gender × Income interaction, *b* = -0.176, *SE* = 0.088, *CI_95%_ =* [-0.352, -0.000], *t*(56) = -2.008, *p* = .050. This interaction was characterized by a non-significant decline in ambivalence toward the rich (vs. poor) with increasing income for men, *b* = -0.146, *SE* = 0.074, *CI_95%_ =* [0.002, -0.295], *t*(56) = -1.972, *p* = .054. This same relationship was non-significant for women, *b* = 0.030, *SE* = 0.047, *CI_95%_ =* [-0.064, 0.123], *t*(56) = 0.636, *p* = .527. This overall pattern is consistent with increasing implicit bias as a function of increasing income in men but not women (see main analyses). All other effects in this analysis of ambivalence toward the rich versus the poor were non-significant, *p* > .13.

**Self-concept centrality.** Like the other models, the self-concept model showed no significant effects. However, there were two non-significant main effects that are worth comment. Should these main effects become reliable in the larger and therefore better powered confirmatory sample, they may provide additional evidence of internal pressure to modulate status bias in high-status individuals. Both gender (men > women) and increasing income appeared to predict increasing agreement with the notion that accepting the poor (vs. rich) is important to one’s self-concept, *b* = -0.540, *SE* = 0.286, *CI_95%_ =* [-1.105, 0.025], *t*(155) = -1.889, *p* = .061, and *b* = -0.267, *SE* = 0.144, *CI_95%_ =* [-0.551, 0.018], *t*(155) = -1.850, *p* = .066, respectively. Effects from all other models were non-significant, *p* > .08.

## Confirmatory Dataset

**Personal evaluations.** In the model examining the participants’ attitudes toward the poor versus rich, we found that women showed greater pro-rich bias than men, *b* = -0.687, *SE* = 0.203, *CI_95%_ =* [-1.085, -0.289], *t*(715) = -3.387, *p* = .001. Additionally, increasing income was associated with reduced explicit pro-rich bias, *b* = -0.252, *SE* = 0.103, *CI_95%_ =* [-0.454, -0.050], *t*(715) = -2.453, *p* = .014. Both main effects were implicated in a significant Gender × Income interaction, *b* = 0.428, *SE* = 0.206, *CI_95%_ =* [0.024, 0.832], *t*(715) = 2.082, *p* = .038, and a significant three-way interaction with education (see Figure S3), *b* = 0.425, *SE* = 0.192, *CI_95%_ =* [0.048, 0.802], *t*(715) = 2.213, *p* = .027. All other effects were non-significant, p > .06.


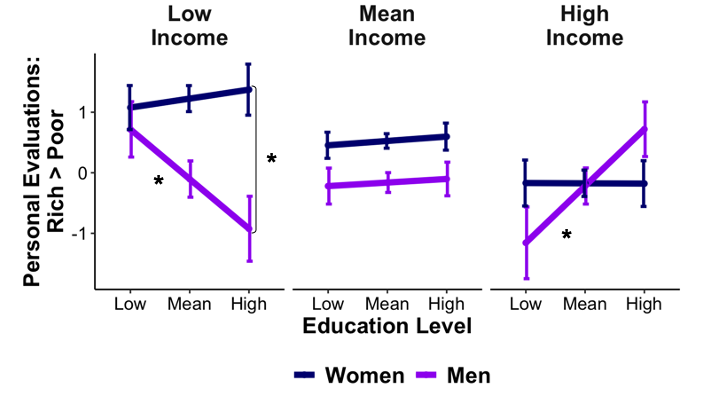


*Figure S3.* Fitted estimates for pro-wealthy explicit bias in personal evaluations as a function of the participant’s gender, education, and income in the confirmatory dataset. Error bars represent standard error for each estimate. All significant simple effects and slopes are indicated with asterisks, *p* < .05.

To decompose the three-way interaction, we tested for significant Gender × Education interactions at low- and high-income levels (± 1.5 *SD*). For completeness, we provide all tests of simple effects to supplement these follow-up analyses (see Table S5). Although both the sign of the interaction coefficient reversed from low to high income, both Gender × Education interaction effects were non-significant: low income, *b* = -0.646, *SE* = 0.345, *CI_95%_ =* [-1.323, 0.031], *t*(715) = -1.872, *p* = .062, high income, *b* = 0.629, *SE* = 0.356, *CI_95%_ =* [-0.070, 1.327], *t*(715) = 1.767, *p* = .078.


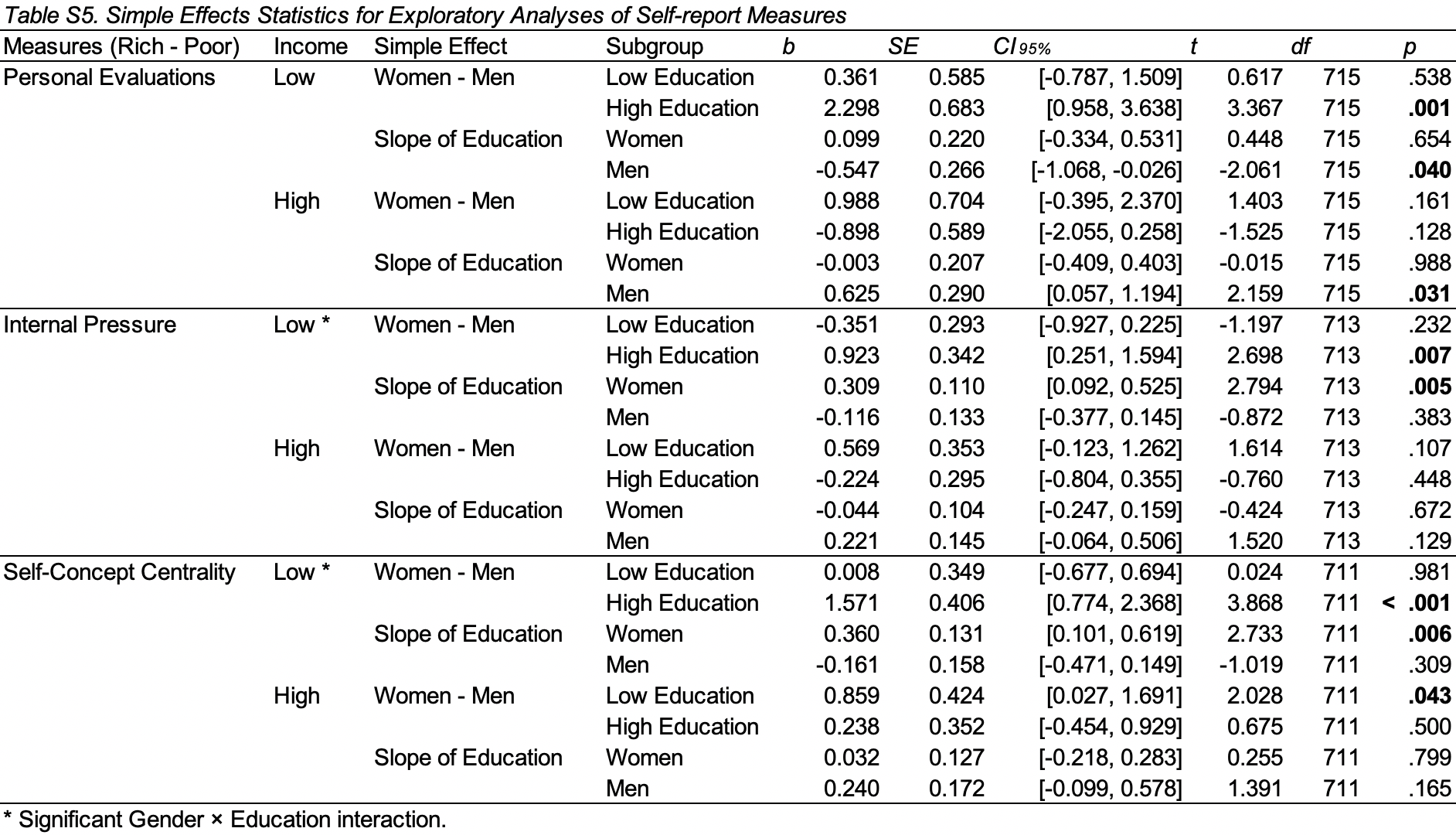


Post hoc inspection of Figure S3, suggested that the three-way interaction between gender, education, and income was driven by a crossover interaction between income and education in men. To further explore this possibility, we tested for significant Income × Education interactions in explicit personal evaluations separately for women and men. These follow-up analyses revealed a significant Income × Education interaction for men, *b* = 0.391, *SE* = 0.153, *CI_95%_ =* [0.091, 0.690], *t*(715) = 2.562, *p* = .011, but not for women, *b* = -0.034, *SE* = 0.117, *CI_95%_ =* [-0.263, 0.195], *t*(715) = -0.291, *p* = .771. Simple slopes of education at each income level indicated that education decreased explicit pro-rich bias for low-income men but increased explicit pro-rich bias for high-income men (see Table S5 for statistics). Simple effects of income at each education level were also consistent with the observed crossover interaction pattern in men; income decreased explicit pro-rich bias for men with low education levels, *b* = -0.625, *SE* = 0.292, *CI_95%_ =* [-1.199, -0.050], *t*(715) = -2.136, *p* = .033, and increased explicit pro-rich bias for men with high education levels, *b* = 0.548, *SE* = 0.274, *CI_95%_ =* [0.010, 1.086], *t*(715) = 2.001, *p* = .046.

**Others’ evaluations.** We found that increasing education was associated with reduced perceptions that others are explicitly biased in favor of the rich, *b* = -0.097, *SE* = 0.037, *CI_95%_ =* [-0.171, -0.024], *t*(716) = -2.601, *p* = .010. All other effects were non-significant, *p* > .07.

**Cultural evaluations.** Men reported that cultural biases favored the rich more than did women, *b* = 0.650, *SE* = 0.261, *CI_95%_ =* [0.139, 1.162], *t*(715) = 2.495, *p* = .013. All other effects were non-significant, *p* > .16.

**Internal pressure.** In the model examining the participants’ self-reported internal pressure to make their evaluations more positive based on others’ wealth, results paralleled findings from the analysis of personal evaluations of the rich and poor. Here, we found that women valued making positive evaluations of the rich (vs. poor) more than did men, *b* = -0.231, *SE* = 0.102, *CI_95%_ =* [-0.430, -0.031], *t*(719) = -2.272, *p* = .023. Additionally, increasing income was associated with reduced explicit pro-rich bias, *b* = -0.103, *SE* = 0.051, *CI_95%_ =* [-0.204. -0.002], *t*(719) = -2.000, *p* = .046. Both main effects were implicated in a significant three-way interaction with education (see Figure S4), *b* = 0.219, *SE* = 0.096, *CI_95%_ =* [0.030, 0.408], *t*(719) = 2.275, *p* = .023. All other effects were non-significant, p > .08.

*
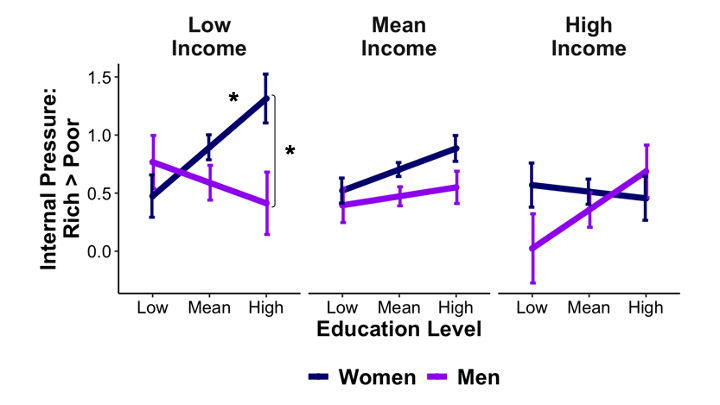
*

*Figure S4.* Fitted estimates for pro-wealthy explicit bias in internal pressure to adjust evaluations based on status. Explicit bias is plotted as a function of the participant’s gender, education, and income in the confirmatory dataset. Error bars represent standard error for each estimate. All significant simple effects and slopes are indicated with asterisks, *p* < .05.

To decompose the three-way interaction, we tested for significant Gender × Education interactions at low- and high-income levels (± 1.5 *SD*). For completeness, we provide all tests of simple effects to supplement these follow-up analyses (see Table S5). We found a significant Gender × Education interaction for low income, *b* = -0.425, *SE* = 0.173, *CI_95%_ =* [-0.764, -0.085], *t*(713) = -2.456, *p* = .014, but not for high income, *b* = 0.265, *SE* = 0.178, *CI_95%_ =* [-0.086, 0.615], *t*(713) = 1.484, *p* = .138.

**Pressure from others and culture, ambivalence.** All predictors for these dependent variables were non-significant, *p* > .06.

**Self-concept centrality.** As in the pilot dataset, women agreed more than men with the notion that accepting the rich (vs. poor) is important to one’s self-concept, *b* = -0.650, *SE* = 0.120, *CI_95%_ =* [-0.886, -0.414], *t*(717) = -5.398, *p* < .001. As for personal values and internal pressure, we also observed here a significant Gender × Income × Education interaction (see Figure S5), *b* = 0.239, *SE* = 0.115, *CI_95%_ =* [0.014, 0.465], *t*(717) = 2.083, *p* = .038. All other effects were non-significant, *p* > .05.


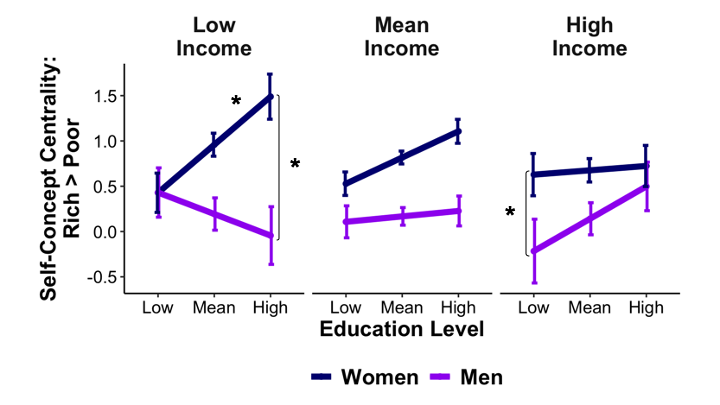


*Figure S5.* Fitted estimates for pro-wealthy explicit bias in centrality to the self-concept of making positive evaluations based on status. Explicit bias is plotted as a function of the participant’s gender, education, and income in the confirmatory dataset. Error bars represent standard error for each estimate. All significant simple effects and slopes are indicated with asterisks, *p* < .05.

To decompose the three-way interaction, we tested for significant Gender × Education interactions at low- and high-income levels (± 1.5 *SD*). For completeness, we provide all tests of simple effects to supplement these follow-up analyses (see Table S5). We found a significant Gender × Education interaction for low income, *b* = -0.521, *SE* = 0.206, *CI_95%_ =* [-0.925, -0.117], *t*(711) = -2.532, *p* = .012, but not for high income, *b* = 0.243, *SE* = 0.115, *CI_95%_ =* [-0.214, 0.628], *t*(711) = 0.967, *p* = .334.

# Supplemental Analyses 2: Adolescent Exclusions

Exploratory analyses were conducted by excluding 14 participants between the ages of 12 and 18. The rationale for this exclusion was that income for adolescents may not accurately reflect their actual material resources due to dependence on parents or guardians. Results for the confirmatory predictions were unchanged after excluding adolescents. We report the full results for these analyses here for completeness.

## Relationships between Independent Variables

The confirmatory dataset sans adolescents provided no evidence of a difference between men and women in terms of income or education, |*t*| < 1.32, *p* > .19. As in the main analyses, we observed a significant correlation between our standardized predictors for income and education *r*(752) = .15, *p* < .001. Because the variance inflation factors (VIF) for all model terms in the omnibus model were below 1.5, this exploratory analysis (like the analysis reported in the main text) was not sufficiently impacted by multicollinearity to warrant orthogonalization of income and education as outlined in our pre-registered analysis plan.

## Confirmatory Analysis

In our analysis of IAT *D* scores as a function of gender, income, education, and all possible interactions between these predictors, the only significant effect was the main effect of gender, *b* = 0.069, *SE* = 0.029, *CI_95%_ =* [0.012, 0.126], *t*(745) = 2.372, *p* = .018. As in the main text, men showed greater implicit pro-rich bias than did women. All other effects were non-significant, *p* > .072. We now turn to the results from our primary pre-registered predictions.

**H1: Gender × Income interaction.** The Gender × Income interaction was non-significant, *b* = -0.024, *SE* = 0.029, *CI_95%_ =* [-0.081, 0.033], *t*(745) = -0.829, *p* = .408. Despite the absence of a formal interaction, we nonetheless followed up on our pre-registered follow-up analyses for men (H1A) and women (H1B). As in the main text, income increased implicit pro-rich bias for women, *b* = 0.037, *SE* = 0.017, *CI_95%_ =* [0.003, 0.071], *t*(745) = 2.162, *p* = .031, but not for men, *b* = 0.013, *SE* = 0.024, *CI_95%_ =* [-0.034, 0.059], *t*(745) = 0.540, *p* = .589.

**H2: Gender × Income × Education interaction.** As in the main text, the predicted three-way interaction was also non-significant, *b* < 0.001, *SE* = 0.028, *CI_95%_ =* [-0.055, 0.054], *t*(745) = -0.017, *p* = .987. Despite the absence of a formal interaction, we again conducted our pre-registered follow-up analyses testing for effects of education level separately for four different participant groups. Effects of education on implicit pro-rich bias were again non-significant for all: low-income women (H2A), *b* = -0.041, *SE* = 0.031, *CI_95%_ =* [-0.102, 0.020], *t*(745) = -1.327, *p* = .185, low-income men (H2B), *b* = 0.011, *SE* = 0.040, *CI_95%_ =* [-0.067, 0.089], *t*(745) = 0.281, *p* = .779, high-income women (H2C), *b* = -0.033, *SE* = 0.029, *CI_95%_ =* [-0.091, 0.024], *t*(745) = -1.134, *p* = .257, and high-income men (H2D), *b* = 0.018, *SE* = 0.042, *CI_95%_ =* [-0.064, 0.099], *t*(745) = 0.425, *p* = .671.
